# The Association Between Mediterranean Diet -Related Health Literacy, Cooking Skills and Mediterranean Diet Adherence in the Spanish Population

**DOI:** 10.3390/nu18020235

**Published:** 2026-01-12

**Authors:** Maria Giulia Casucci, Júlia Muñoz-Martínez, Begoña Caneda-Ferrón, Blanca Salinas-Roca, Alicia Orta-Ramirez, Eulàlia Vidal, Míriam Rodríguez-Monforte, Inês Medeiro da Costa, Vânia Costa, Sofia Renzi, Elena Carrillo-Álvarez

**Affiliations:** 1 Global Research on Wellbeing (GRoW) Research Group, Blanquerna School of Health Science, Ramon Llull University, Padilla, 326-332, 08025 Barcelona, Spain; juliamm1@blanquerna.url.edu (J.M.-M.); begonaangelicacf@blanquerna.url.edu (B.C.-F.); eulaliavg@blanquerna.url.edu (E.V.); miriamrm@blanquerna.url.edu (M.R.-M.); elenaca@blanquerna.url.edu (E.C.-Á.); 2 Dietetics and Nutrition Department, Escola Superior de Saúde de Lisboa, Instituto Politécnico de Lisboa (IPL), Av. D. João II, Lote 4.69.01, Parque das Nações,1990-096 Lisboa, Portugal; ines.costa@estesl.ipl.pt; 3 H&TRC—Health and Technology and Research Center, ESSL—Escola Superior de Tecnologia da Saúde de Lisboa, Av. D. João II, Lote 4.69.01, Parque das Nações, 1990-096 Lisboa, Portugal; vania.costa@estesl.ipl.pt; 4 Biochemistry Laboratory, School of Biosciences and Veterinary Medicine, Università di Camerino, Via Gentile III Da Varano, 62032 Camerino, MC, Italy; sofia.renzi@unicam.it

**Keywords:** Mediterranean diet, cooking skills, health literacy, nutrition education, adolescents, life-course perspective, dietary behaviour

## Abstract

**Background/Objectives:** Even with solid proof of its benefits for cardiovascular health and metabolism, adherence to the Mediterranean Diet (MD) in Spain has noticeably declined in recent years. The socioeconomic changes occurring in recent decades have prompted shifts in cooking habits and in how food is socially experienced, particularly among children and adolescents. The MD is more than just food: it is a cultural tradition and a lifestyle, rich in food and cooking skills, and food wisdom passed down over generations. When these practices fade, it affects both health and the environment, making them vital components in strengthening support for food knowledge, cooking abilities, and a healthier lifestyle. Considering these shifting dietary patterns and the growing need for targeted educational strategies, the present study aimed to investigate the association between cooking skills, MD-related health literacy, and adherence to the Mediterranean Diet across different developmental stages: childhood, adolescence, and adulthood in a sample of the Spanish population. Additionally, a secondary objective was to identify potential critical windows for intervention based on the strength of these associations. **Methods:** This cross-sectional study included 832 Spanish participants grouped by age: children and early adolescents (*n* = 408), older adolescents (n = 136), and adults (n = 288). Cooking skills were assessed using CooC11 for children and FCSk for older groups. Adults also completed Lit_MEDiet to assess MD-related health literacy. Adherence was measured with KIDMED (children/adolescents) and MEDAS (adults). Spearman correlations and standardized linear regressions were used. All statistical tests were two-sided, and statistical significance was set at *p* < 0.05. **Results:** In children, no significant association was found between cooking skills (CooC11) and KIDMED scores (β = 0.008; *p* = 0.875). Among adolescents, a strong positive association emerged between FCSk and KIDMED (β = 0.313; *p* < 0.001; ρ = 0.371), indicating a large, standardized effect and suggesting that this stage is particularly sensitive to food skills. In adults (18+), both food and cooking skills (FCSk) (β = 0.189; *p* = 0.001) and MD-related health literacy (Lit_MEDiet) (β = 0.187; *p* = 0.004) were moderately associated with MEDAS scores. **Conclusions:** These findings suggest that mid-adolescence could represent a favourable developmental window where food skills may hold potential to influence positive dietary behaviours. Regarding adults, the results indicate that combining practical and educational components appears to beneficial for dietary quality. Overall, this study supports the relevance of age-tailored public health strategies to potentially enhance long-term adherence to the Mediterranean Diet.

## 1. Introduction

In Spain, the Mediterranean Diet (MD) is more than a pattern of ingredients: it is a ritual of belonging, rooted in shared meals, seasonal produce, and intergenerational knowledge [1]. This heritage is currently challenged by the significant decline in adherence to the MD over recent decades. This trend is driven by a rapid nutritional transition characterized by the westernization of diets and a shift towards energy-dense, ultra-processed foods [1,2,3,4]. Recent population evidence further suggests a marked social gradient, with lower adherence among adolescents and young adults and disparities by place and resources, including poorer diet quality in urban compared with rural settings and in socioeconomically disadvantaged households, where MD adherence is lower [3,5,6]. Amidst these pressures, perceived time scarcity and the omnipresence of ultra-processed foods have transformed cooking from a traditional practice into a daily burden. Consequently, as culinary traditions fade, so does the transmission of the social and environmental values that historically sustain Mediterranean food cultures [1].

This erosion of everyday cooking and commensality is not merely a cultural concern. It represents a behavioural and educational challenge: the gradual loss of competencies that enable individuals to engage critically and autonomously with food. Among these, three distinct but interrelated dimensions stand out: cooking skills, food skills, and food literacy. To avoid conceptual ambiguity, cooking skills are defined here as the mechanical and physical techniques required to prepare ingredients and utilize kitchen equipment (e.g., peeling, chopping, mixing, heating) [7]. Food skills, conversely, encompass the broader organizational and resource management capabilities necessary to plan and provision dietary intake, including budgeting, shopping strategies, and the technical reading of food labels [7]. Finally, food literacy is framed as a higher-order construct that integrates these functional abilities with the critical cognitive understanding of the diet’s health, social, and environmental dimensions needed to make informed lifestyle choices, such as seasonality and sustainability [8,9]. Together, these competencies constitute what has been described as “food agency”, i.e., the capacity to act purposefully and reflectively within food environments shaped by social and structural constraints [4,10,11].

A growing body of evidence shows that such competencies contribute to healthier and more sustainable diets. Adults with higher cooking competence and health literacy tend to cook more frequently at home, consume more fruits and vegetables, and exhibit greater adherence to the MD [12,13,14]. In Mediterranean populations, cooking skills alone may explain up to one-quarter of the variance in adherence to this dietary model [15,16]. Among adolescents, stronger culinary abilities have been associated with lower consumption of ultra-processed foods and better adherence to MD-consistent behaviours [17,18,19]. Yet most studies have focused on adults or university students, leading to a limited understanding of how these competencies develop across the life course and at which point they exert the greatest influence.

A life-course perspective provides a crucial lens for addressing this gap. Food-related skills and literacy evolve alongside developmental, social, and cognitive changes. During childhood, food choices are largely shaped by parents, schools, and environmental exposure, leaving little room for independent decision-making [20,21]. In adolescence, however, growing autonomy and identity formation make this stage a particularly sensitive and malleable period for behavioural change [22,23,24]. The World Health Organization and European frameworks, such as Health Promoting Schools and Farm to Fork, identify adolescence as a “teachable moment” for embedding food competencies that extend into adulthood [25,26]. In contrast, adults tend to consolidate established habits influenced by time availability, work patterns, and motivational factors [5,14]. The MD offers a particularly suitable lens through which to examine how knowledge, skills, and everyday practices interact across the lifespan [1,27]. As a dietary pattern strongly embedded in cultural identity, sustainability principles, and social organization, it illustrates how food-related behaviours are shaped by both individual capacities and broader environments [28,29].

Because food competencies evolve with autonomy and cognitive development, their associations with dietary behaviour are likely to differ across childhood, adolescence, and adulthood; identifying when cooking skills and MD-related health literacy are most strongly linked to MD adherence can inform developmentally tailored public health strategies [30,31,32]. Guided by this perspective, the present study utilizes a cross-sectional analysis of harmonized data from the PRIMA-funded Tool4MEDLife project [33]. Leveraging age-specific validated measures, we aimed to investigate the associations between cooking skills, MD-related food literacy, and adherence to the Mediterranean Diet across three distinct developmental stages: childhood, adolescence, and adulthood. A secondary objective was to identify potential critical windows for intervention based on the strength of these associations.

## 2. Materials and Method

### 2.1. Design and Participants

A descriptive cross-sectional study was conducted in a sample of 832 children, adolescents, and adults in Spain to examine the association between cooking skills, MD-related literacy, and MD adherence across different developmental stages.

Participants were recruited using a convenience sampling strategy to maximize data quality and participant engagement through supervised administration in structured educational and community settings. As a non-probability approach, this strategy may limit representativeness and generalizability. Participants were eligible if they resided in Spain at the time of data collection and were able to independently comprehend and complete the questionnaire in Spanish or Catalan. The final sample was stratified into three age groups—children (8–14 years), adolescents (15–17 years), and adults (≥18 years)—reflecting the age ranges for which validated, age-appropriate instruments were available within the project. This stratification was designed to capture distinct developmental stages of food agency: children are characterized by substantial parental dependence, adolescents represent a critical window for emerging autonomy, and adults correspond to the stage where dietary habits are typically consolidated. Individuals younger than eight years were excluded, as independent comprehension of the questionnaires could not be ensured.

Prior to participation, all individuals received information covering the study aims, confidentiality, privacy, and the anonymous nature of data collection. No directly identifying information was collected. For adults, informed consent was obtained electronically. For underage participants, written informed consent was obtained from parents or legal guardians, and minors also provided additional written assent. All procedures complied with the ethical principles of the Declaration of Helsinki and were approved by the Institutional Review Board of Ramon Llull University, Barcelona, Spain (CER URL_2023_2024_014]. The study was reported following the STROBE guidelines [34] for cross-sectional studies, and the completed checklist is provided as Supplementary Materials File S1.

### 2.2. Data Collection and Study Variables

Data collection was conducted between May and December 2024 following standardized procedures across all study settings. All questionnaires were integrated into a single survey administered via the Qualtrics platform. For children and adolescents, data were collected during supervised, school-based group sessions. Children completed paper-based questionnaires, with trained researchers providing standardized instructions and support to ensure comprehension. When paper questionnaires were used, responses were subsequently entered into the Qualtrics platform by the research team. Adolescent and adult participants completed the survey online. All instruments were administered in Spanish or Catalan according to participant preference.

Sociodemographic variables were assessed through dedicated age-specific modules. Across all age groups, sociodemographic information included age and sex. Additional variables for school-aged participants included region, type of school (public or private), parental education and parental employment status, household composition, and school canteen attendance. For adults, further information included nationality and country of birth, highest educational attainment, employment status, and household composition.Self-perceived socioeconomic status was assessed in adolescents and adults using an ordinal self-report item. For children, simplified sociodemographic questions were administered directly. In this age group, socioeconomic status was additionally assessed using the Family Affluence Scale III (FAS III) [35], a four-item measure of material affluence developed within the Health Behaviour in School-aged Children survey. The total FAS III score ranges from 0 to 9, with higher scores indicating greater family affluence. As FAS III was implemented for descriptive purposes only, it was summarized as a continuous variable and was not included in the regression analyses.

Health and nutritional status variables were collected using age-adapted versions of the European Health Interview Survey (EHIS) [36], tailored to each participant group. For adolescents and adults, the EHIS module collected self-reported information on perceived health status (variable HS1), lifestyle, the presence of long-term physical or mental health conditions (variable HS2), and anthropometric data including height and weight (variables BM1 and BM2). For adolescents, a shortened EHIS-based module was self-administered and collected information on the presence of long-term physical or mental health conditions (variable HS2) and anthropometric data (variables BM1 and BM2).

Adherence to the MD was assessed using age-specific validated instruments. In children and adolescents, adherence was measured using the KIDMED 2.0 index [37]. The instrument comprises 16 dichotomous items reflecting dietary behaviours consistent or inconsistent with MD principles. Positive behaviours are scored as +1 and negative behaviours as −1, yielding a total score ranging from −16 to +12, with higher scores indicating greater adherence. In adults, adherence to the Mediterranean Diet was assessed using the Mediterranean Diet Adherence Screener (MEDAS) [38]. Within the Tool4MEDLife framework, the instrument was refined to improve measurement precision. First, to reduce errors in portion size estimation, photographic visual aids derived from the Manual Fotográfico de Quantificação de Alimentos were incorporated [39]. Second, vegetable consumption was captured through two separate items to account for preparation method: participants reported their intake of cooked vegetables (target of at least 2 servings per day; 1 serving = 200 g) and raw vegetables (target of at least 1 serving per day). To maintain consistency with the original MEDAS scoring algorithm, these two items were harmonized during analysis by assigning a score of 1 if the participant met the threshold for either cooked or raw vegetables, thereby preserving the standard total score range (0 to 14).

Cooking skills were assessed using age-appropriate instruments reflecting participants’ autonomy in food preparation. In children and early adolescents, cooking competence was measured using the Competencia Culinaria 11 (CooC11©) [40], a 10-item questionnaire supported by engaging illustrations of peer models for self-assessment of perceived competence in everyday culinary tasks. Responses were rated on a 5-point Likert scale, and a composite score was derived (range 0 to 50), with higher values indicating greater perceived cooking competence. In the present sample, the scale demonstrated high internal consistency (Cronbach’s alpha = 0.864). In adolescents and adults, food and cooking skills were assessed using the comprehensive Food and Cooking Skills Questionnaire (FCSk) [4]. The instrument assesses diverse competencies including preparation techniques, planning, and food management. Items were rated on a 7-point Likert scale; a maximum score of 228 and 245 could be obtained for the adolescent and adult versions, respectively. The psychometric properties of the FCSk in this study were excellent, showing strong internal consistency (Cronbach’s alpha = 0.887).

MD-related health literacy was assessed among adult participants using the Lit_MEDiet [32], an instrument previously designed and specifically validated within the framework of the Tool4MEDLife project (manuscript in preparation). Unlike general food literacy tools, this instrument was developed to specifically capture competencies related to the Mediterranean lifestyle. For the purpose of the regression analysis, a re-coded summary score (range 0 to 4) was derived to capture the key dimensions of MD knowledge, where higher scores indicate better MD-related health literacy. Internal consistency for the Spanish adult sample was robust (Cronbach’s alpha = 0.835)

Questionnaires originally validated in Spanish, including KIDMED 2.0 and MEDAS, were used in their validated versions. Instruments not previously available in Spanish, including CooC11, FCSk, and Lit_MEDiet, underwent a standard translation and cultural adaptation process. Two members of the research team independently translated the original English versions into Spanish, and the national research team reviewed the translations to ensure linguistic clarity, cultural relevance, and age-appropriate wording. The Spanish versions were subsequently back-translated into English by an external bilingual translator unfamiliar with the original instruments, and discrepancies were resolved by consensus. Average completion time was approximately 20 to 30 min for adolescents and adults and was longer for younger participants due to guided administration. Completed questionnaires were reviewed for missing or inconsistent responses and coded using a harmonized protocol developed by the research team. Data entry was performed independently by two researchers; discrepancies were resolved by consensus, and random double-entry checks were conducted on 10 percent of the dataset to ensure data accuracy and integrity.

### 2.3. Statistical Analysis

Descriptive statistics (mean ± standard deviation for continuous variables; frequencies and percentages for categorical variables) were calculated to characterize the sample. Normality of the data distribution was assessed using the Shapiro–Wilk test and visual inspection of Q-Q plots. Data quality procedures included screening for duplicate entries and inconsistent response patterns. Missing data were handled using listwise deletion; no imputation methods were applied.

Continuous variables were screened for extreme outliers, defined as values exceeding three standard deviations from the mean, which were excluded from the final analysis.

Specific predictor–outcome associations were defined by developmental stage: for children (8–14 years), cooking competence (CooC11) was examined in relation to KIDMED; for adolescents (15–17 years), food and cooking skills (FCSk) were analyzed against KIDMED; and for adults (≥18 years), both FCSk and MD-related literacy (Lit-MEDiet) were examined as predictors of MEDAS scores. Bivariate associations were assessed using Spearman’s rank correlation coefficients (ρ) due to the non-normal distribution of the variables.

For exploratory modelling, univariable Ordinary Least Squares (OLS) regression models were fitted on standardized predictor and outcome scores to derive standardized β coefficients, enabling direct comparison of association magnitudes across developmental stages. OLS was considered appropriate given its robustness to moderate deviations from normality in samples exceeding 100 observations. Multivariable adjustment was not applied because key sociodemographic and socioeconomic indicators were not measured in a fully harmonized, conceptually equivalent way across age groups (for example, parental education and household indicators in minors versus individual education and employment in adults); including non-comparable covariates would have reduced interpretability and comparability across stages.

Potential confounders such as socioeconomic status and parental education were collected (age-specifically) and examined descriptively but were not included in regression models for the reasons above.

Sociodemographic variables were therefore summarized descriptively, and the present estimates should be interpreted as exploratory associations rather than causal effects. All analyses were performed using Python 3.10 (Pandas/Scipy/Statsmodels libraries), with significance set at *p* < 0.05.

## 3. Results

A total of 832 individuals participated in this study. After data cleaning and exclusion of missing values, the final analytical sample comprised 382 children and early adolescents (8–14 years), 136 adolescents (15–17 years), and 288 adults (18+ years). As shown in Table 1, the sex distribution was balanced in the younger groups, whereas the adult sample was predominantly female (74.0%). Descriptive statistics for the main study variables, including mean (SD), median (IQR), and observed ranges, are reported in Table 2. Among adults, the Lit-MEDiet module was completed by 233 participants; the remaining 55 adults had missing items in this specific section and were therefore excluded from analyses involving this variable (measure-specific listwise deletion).

Adherence to the Mediterranean Diet (KIDMED/MEDAS) showed variability across age groups. Regarding culinary skills, scores for both cooking competence (CooC11) in children and food and cooking skills (FCSk) in adolescents and adults exhibited a wide distribution covering the entire theoretical scale of the instruments, reflecting substantial heterogeneity in culinary proficiency within the sample.

The relationship between food-related competencies and MD adherence exhibited a distinct pattern in different developmental ages (see Table 3 and Table 4). All associations reported below are univariable and should be interpreted as exploratory; age-specific sociodemographic factors may confound the observed relationships.

In children aged 8–14 years, Spearman correlation analysis showed no significant association between cooking competence (CooC11) and Mediterranean Diet adherence (KIDMED) \ρ = −0.018, *p* = 0.733), indicating a negligible relationship. Similarly, univariable linear regression indicated a non-significant association (β = 0.008, *p* = 0.875). This null association likely reflects the limited food agency of this age group, whose dietary choices are predominantly shaped by parental control rather than individual skills.

In contrast, among adolescents aged 15–17 years, food and cooking skills (FCSk) were positively and significantly associated with MD adherence. Spearman analysis revealed a moderate to large correlation (ρ = 0.371, *p* < 0.001). Univariable linear regression confirmed this strong association (β = 0.313, 95% CI: 0.012 to 0.038, *p* < 0.001), with skills explaining nearly 10% of the variance in diet quality (R^2 = 0.098).

**Figure 1:**
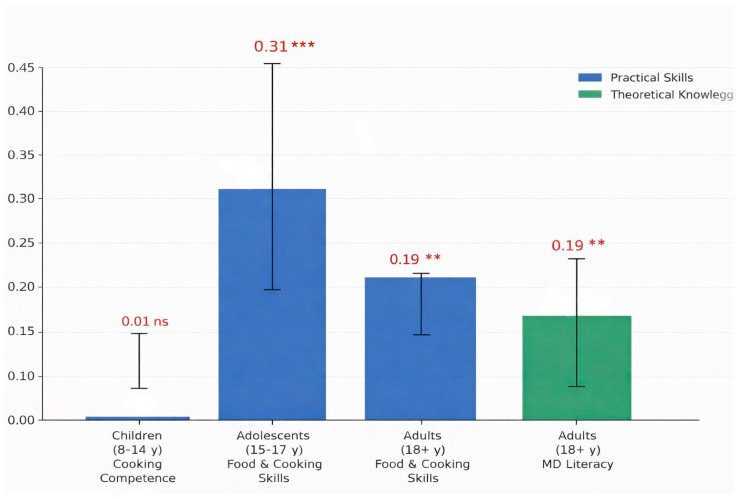
Standardized associations between food-related competencies and MD adherence across developmental stages. Bars represent standardized linear regression coefficients (β), allowing for comparison of effect sizes across different instruments. Predictors include cooking competence (CooC11) for children (8–14 y), food and cooking skills (FCSk) for adolescents (15–17 y) and adults (18+ y), and MD-related health literacy (Lit-MEDiet) for adults. The outcome variable is MD adherence (assessed via KIDMED index for minors and MEDAS for adults). Error bars represent 95% confidence intervals. Significance levels: *** *p* < 0.001 and ** *p* < 0.01; ns = non-significant (*p* > 0.05).

In adults (18+ years), both cooking skills (β = 0.189, *p* = 0.001) and MD-related literacy (β = 0.187, *p* = 0.004) were significantly associated with MEDAS scores, although correlation magnitudes were small (ρ = 0.174 and ρ = 0.186, respectively). Furthermore, exploratory analysis revealed a significant positive correlation between FCSk and Lit-MEDiet scores (ρ = 0.245, *p* < 0.001), suggesting that practical and cognitive food competencies are interrelated in the adult population. Figure 1 shows a summary of the results.

## 4. Discussion

This study examines age-stratified associations in a large Spanish sample using harmonized tools validated across age groups. It is, to our knowledge, among the first to examine, from a life-course perspective, the associations between cooking skills, MD-related health literacy, and adherence to the MD across childhood, adolescence, and adulthood in a Spanish sample. Our approach builds on the understanding that dietary behaviours result from the interaction between individual competencies and layered contexts, where agency, family practices, and everyday constraints shape whether knowledge and skills translate into consistent adherence [4,5,6,7,8,9,18,19,20]. In this study, we observed a clear age gradient: no significant association in children, a moderate yet significant association in adolescents, and moderate but significant associations in adults.

In this regard, our results suggest that the association between food and cooking skills and MD adherence is particularly pronounced in adolescence. Adolescents with higher cooking-related competence reported higher adherence to the MD. The standardized β of 0.313 indicates a medium-to-large effect size (according to Cohen’s guidelines), representing a substantial effect relative to other behavioural predictors of diet quality and highlighting the practical importance of these skills during this developmental window. Notably, this association is considerably stronger than that observed in our adult sample (β= 0.189), highlighting that adolescence represents a critical developmental window where these skills play a more decisive role in dietary quality compared to later stages of life.

This finding is consistent with the characterization of adolescence as a developmental stage marked by increasing autonomy, identity construction, and heightened sensitivity to social norms and environments [22,41]. As opportunities to make or negotiate food decisions expand, practical competencies such as planning, preparing meals from basic ingredients, and navigating food environments may become decisive determinants of dietary patterns [10,42,43]. Evidence from school and community experiences suggests that experiential approaches, including cooking-based education and food-related activities, can improve food-related competencies and diet quality, supporting adolescence as a potentially sensitive window for intervention [40,44,45,46,47]. From a public health standpoint, this aligns with frameworks promoting schools and community settings as strategic entry points for early prevention of diet-related non-communicable diseases and healthy habit formation [21,23,41,48].

In adults, both food and cooking skills and MD-related health literacy were significantly associated with MD adherence, with comparable effect sizes. This pattern suggests complementarity between practical and conceptual dimensions: literacy may support informed decision-making, while cooking skills enable implementation under real-world constraints such as time pressure, budget considerations, household characteristics, and competing responsibilities [4,5,6,7,11,12,13].

It is likely that socioeconomic factors, urbanization, and cultural shifts moderate these associations; for instance, individuals with limited resources may rely more heavily on practical cooking skills to maintain a healthy diet amidst the increasing availability of ultra-processed foods [9,14,42]. Consequently, adult interventions, particularly those targeting high-risk or low-literacy subgroups, may be most effective when combining practical skill-building with actionable nutrition information to maximize metabolic and cardiometabolic health benefits [10,11,12,13].

The lack of association in children is unsurprising. During childhood, dietary patterns are largely mediated by parents, caregivers, and the school food environment, as children have limited control over procurement, meal planning, and household rules [18,20,23]. Even when children report confidence in simple tasks, this perceived competence may not translate into meaningful influence over what is eaten at home or at school. Accordingly, childhood interventions may be more relevant for shaping exposure, familiarity, and positive attitudes toward foods and preparation practices, which can later support engagement when autonomy increases during adolescence [29,40,45,46,47].

Taken together, these findings emphasize that the MD should be understood not only as an aggregate of food choices but also as a cultural and social practice sustained through shared routines, intergenerational transmission, and community norms [1,2,26,27,28]. This perspective reinforces the importance of designing nutrition education strategies that leverage collective contexts such as schools, community centres, and intergenerational spaces, where modelling, participation, and social reinforcement can strengthen both motivation and competence [23,29,40,43,44,45,46,47,48]. Within this framework, the present results provide empirical support for the educational toolkits developed in the PRIMA-funded Tool4MEDLife project, which integrate experiential and structured resources across age groups [33]. Our age-stratified pattern suggests that behavioural impact may be particularly high when toolkits prioritize adolescents with skills-based modules, while adult-directed resources should balance practical and informational components to accommodate heterogeneous needs and constraints [4,5,6,7,12,22,41].

Several limitations should be acknowledged. First, the cross-sectional design prevents causal inference and does not rule out reverse causation, whereby individuals with higher MD adherence may be more inclined to engage in cooking or seek MD-related information [10,11,12]. Second, convenience sampling may limit generalizability. In particular, the adult sample was predominantly female (74%); while this reflects traditional gender roles in domestic food preparation, it may limit the applicability of findings to the male population, warranting caution in interpretation. Third, reliance on self-reported data may have introduced social desirability biases [34]. Specifically, regarding the null association in children, unmeasured factors such as parental reporting bias or the disconnect between child competence and actual household decision-making power may have obscured potential relationships [34]. Fourth, relevant contextual determinants such as parental influence, frequency of cooking at home, household food rules, school meal characteristics, and neighbourhood food environments were not captured; these factors may condition how competencies translate into adherence, particularly in childhood [18,19,20,23].

Despite these limitations, this study has several strengths. It applies a comparative, life-course design using harmonized procedures and validated tools within the Tool4MEDLife framework, allowing for age-specific measurement while enabling cross-group comparison [19,33,37,38]. Moreover, the use of standardized coefficients supports interpretation of relative effect sizes across distinct constructs, strengthening the main conclusion that adolescence is the stage in which the skills–adherence link is most pronounced [22,41].

Future research should build on these findings through longitudinal and intervention studies to test whether improvements in cooking skills and MD-related health literacy during adolescence predict sustained MD adherence into adulthood [41,49,50]. Additionally, comparative analyses across Mediterranean countries and diverse socioeconomic contexts could clarify how structural factors, cultural norms, and food environments shape the observed gradient, particularly considering documented dietary transitions in the Mediterranean area [1,2,3]. From a public health standpoint, the results support prioritizing adolescent-focused cooking and nutrition education as a potentially high-impact prevention strategy, while reinforcing the need for integrated adult approaches combining literacy and skill-building [20,23,41,48]. Ultimately, enabling individuals to understand, value, and apply the MD throughout their life is crucial not only for preserving cultural heritage but also for improving cardiometabolic health outcomes in the broader population [1,2,26,27,28,51,52].

## 5. Conclusions

By adopting a life-course perspective, this study highlights how the influence of food-related competencies evolves from childhood to adulthood. We provide evidence that mid-adolescence is a pivotal period where practical cooking skills are most strongly associated with adherence to the Mediterranean Diet, showing a substantial standardized effect (beta = 0.313) compared to other life stages. This finding has significant implications for public health policy and practice. It suggests that resources should be strategically allocated to programmes that foster hands-on culinary skills in secondary school students. Such interventions, timed to coincide with this critical developmental window, hold the potential to instil lifelong competencies that empower individuals to make healthier food choices, thereby promoting long-term health and well-being. For adults, interventions should continue to support skill maintenance while also bolstering MD-related health literacy to address the multifaceted nature of adult eating behaviours. Ultimately, fostering these competencies promotes long-term well-being, thereby supporting not only individual health but also the preservation of Mediterranean food culture and sustainable dietary practices.

## Figures and Tables

**Table 1:** Sociodemographic characteristics by age group.

Characteristic	Children and Early Adolescents (8–14 Years) ᵃ	Adolescents (15–17 Years) ᵇ	Adults (18+ Years)
Total Study Sample (N)	407	135	288
Valid n for sociodemographics	311 ᶜ	117	232
Age (years), Mean (SD)	11.68 (2.85)	15.82 (0.72)	55.0 (13.0)
Gender, n (%)			
Female	175 (56.3)	72 (61.5)	172 (74.1)
Male	124 (39.9)	42 (35.9)	60 (25.9)
Other/Prefer not to say	12 (3.8)	3 (2.6)	—
Nationality, n (%)			
Spanish	274 (89.2)	106 (90.6)	226 (97.4)
Other	33 (10.8)	11 (9.4)	6 (2.6)
Household size, Mean (SD)	4.04 (0.98)	4.06 (1.08)	2.63 (1.21)
Socioeconomic Indicators	(Maternal Report) ᵈ	(Maternal Report)	(Self-Report)
Education Level, n (%)			
Primary or less	22 (9.4)	3 (2.6)	3 (1.3)
Secondary (ESO)	41 (17.5)	22 (18.8)	20 (8.7)
Upper Sec./Vocational	83 (35.5)	69 (59.0)	23 (10.0)
Tertiary (University)	87 (37.2)	15 (12.8)	184 (80.0)
Missing/Not Collected	174	26	—
Employment Status, n (%)			
Employed	159 (67.9)	81 (80.2)	144 (63.0)
Unemployed	7 (3.0)	1 (1.0)	6 (2.6)
Homemakers/Other	68 (29.1)	19 (18.8)	80 (34.7)

Note: Values are presented as n (%) for categorical variables and mean (SD) for continuous variables. Percentages are calculated based on valid responses excluding missing values. ᵃ Children and Early Adolescents group: This category combines children (8–10 years, recruited from primary schools) and early adolescents (11–14 years, recruited from lower secondary schools). While age and gender data are available for the entire group, detailed parental socioeconomic data were not collected in the Children’s module (n = 155). Consequently, the SES indicators presented in this column predominantly reflect the early adolescents subsample. ᵇ Adolescents group: this includes participants aged 15–17 years recruited from upper secondary schools. ᶜ Valid n: this reflects the number of participants with complete data for general sociodemographic variables (age, gender, nationality). ᵈ Proxy reporting: For children, early adolescents, and adolescents, maternal education and employment were used as proxy indicators for family socioeconomic status.

**Table 2:** Descriptive statistics of KIDMED, MEDAS, CooC11, FCSk, and Lit-MEDiet.

Developmental Stage	Variable	n ^a^	Mean (SD)	Median [IQR]	Observed Range (Min–Max)	Theoretical Range
Children and Early Adolescents	MD Adherence (KIDMED)	382	6.30 (3.01)	6.00 [4.00–8.00]	−1 to +12	−16 to +12
(8–14 years)	Cooking Competence (CooC11)	382	26.21 (13.09)	24.00 [12.00–33.00]	0 to 50	0 to 50
Adolescents	MD Adherence (KIDMED)	136	6.20 (3.00)	6.00 [4.00–8.00]	−1 to +12	−16 to +12
(15–17 years)	Food and Cooking Skills (FCSk)	136	132.11 (39.02)	132.00 [93.50–153.50]	0 to 228	0 to 228
Adults	MD Adherence (MEDAS)	288	8.21 (2.31)	8.00 [7.00–10.00]	2 to 14	0 to 14
(18+ years)	Food and Cooking Skills (FCSk)	288	153.09 (35.08)	149.50 [118.25–172.00]	0 to 238	0 to 245
	MD Health Literacy (Lit-MEDiet)	233 ^b^	117.16 (17.29)	116.00 [108.00–129.00]	74 to 148	0 to 150

Notes: SD = standard deviation; IQR = interquartile range (25th–75th percentile). ^a^ Represents the final analytical sample included in the descriptive analysis. ^b^ The sample size for Lit-MEDiet is lower (n = 233) due to measure-specific missing data in this voluntary survey module.

**Table 3:** Spearman correlations between cooking skills, Mediterranean Diet-related literacy, and Mediterranean Diet adherence by age group.

Age Group	Variables	ρ (rho)	*p*-Value
Children (8–14 y)	CooC11–KIDMED	−0.018	0.733
Adolescents (15–17 y)	FCSk–KIDMED	0.371	<0.001 ***
Adults (18+ y)	FCSk–MEDAS	0.174	0.003 **
	Lit-MEDiet–MEDAS	0.186	0.004 **
	FCSk–Lit-MEDiet *ª*	0.396	<0.001 ***

Notes: Significance levels: ** *p* < 0.01 and *** *p* < 0.001. *ª* Exploratory correlation between the two predictor variables (skills and literacy) in the adult subsample.

**Table 4:** Univariable linear regression models predicting Mediterranean Diet adherence by age group.

Age Group	Predictor	Outcome	Std. β	Unstandardized b	95% CI for b	R2	*p*-Value	n
Children (8–14 y)	CooC11	KIDMED	0.08	0.02	−0.021 to 0.024	<0.001	14.35	382
Adolescents (15–17 y)	FCSk	KIDMED	5.13	0.25	0.012 to 0.038	1.38	<0.001 ***	136
Adults (18+ y)	FCSk	MEDAS	3.09	0.12	0.005 to 0.020	0.36	0.001 **	288
	Lit-MEDiet	MEDAS	3.07	0.25	0.008 to 0.042	0.35	0.004 **	233

Note: standardized beta coefficient (β); b = unstandardized coefficient; CI = confidence interval. All models are unadjusted with univariable linear regressions estimated via OLS. Significance levels: ** *p* < 0.01 and *** *p* < 0.001.

## Data Availability

The data presented in this study are available on request from the corresponding author. Aggregated, anonymized data are also part of the PRIMA Tool4MEDLife consortium repository.

## Supplementary Materials

The following supporting information can be downloaded at https://www.mdpi.com/article/10.3390/nu18020235/s1, File S1: STROBE Statement—Checklist of items that should be included in reports of cross-sectional studies; File S2: Study questionnaires (English translation of data collection instruments: CooC11, FCSk, and Lit-MEDiet); File S3: Python script used for statistical analysis.
