# Supplementary material for: The Association Between Mediterranean Diet -Related Health Literacy, Cooking Skills and Mediterranean Diet Adherence in the Spanish Population
Source: Nutrients. 2026 Jan 12;18(2):235. doi: 10.3390/nu18020235 (PMC12845093; doi:10.3390/nu18020235)
Supplement: Supplementary file 1 [file nutrients-18-00235-s001.zip › File 1 – STROBE checklist for cross-sectional studies.pdf]

STROBE Statement—Checklist of items that should be included in reports of cross-sectional studies

Section	Item No	Recommendation	Reported on Page / Location
Title and abstract	1(a)	Indicate the study's design with a commonly used term in the title or the abstract.	p. 1 (Title: "...cross-sectional study...")
Title and abstract	1(b)	Provide in the abstract an informative and balanced summary of what was done and what was found.	p. 1 (Abstract)
Introduction	2	Explain the scientific background and rationale for the investigation being reported.	pp. 2–3 (Introduction: paragraphs 1–2)
Introduction	3	State specific objectives, including any prespecified hypotheses.	p. 4 (Objectives section)
Methods	4	Present key elements of study design early in the paper.	p. 5 (Methods 2.1: "This cross-sectional study...")
Methods	5	Describe the setting, locations, and relevant dates, including periods of recruitment, exposure, follow-up, and data collection.	p. 5 (Methods 2.1: "...conducted between May and December 2024...")
Methods	6(a)	Give the eligibility criteria, and the sources and methods of selection of participants.	p. 6 (Methods 2.1: Eligibility criteria and recruitment described)
Methods	6(b)	Cohort study—Give methods of follow-up. Case-control study—Give matching criteria... Cross-sectional study—N/A	N/A (Cross-sectional study)
Methods	7	Clearly define all outcomes, exposures, predictors, potential confounders, and effect modifiers.	pp. 6–7 (Methods 2.2: "Data Collection and Study Variables")
Methods	8*	For each variable of interest, give sources of data and details of methods of assessment (measurement).	p. 7 (Methods 2.2: CooC11, FCSk, Lit-MEDiet, KIDMED, MEDAS)
Methods	9	Describe any efforts to address potential sources of bias.	p. 8 (Methods 2.3: "...random double-entry checks conducted...")
Methods	10	Explain how the study size was arrived at.	p. 6 (Methods 2.1: Final sample size n=832)
Methods	11	Explain how quantitative variables were handled in the analyses. Describe groupings.	p. 8 (Methods 2.3: "All variables were z-standardised..."; age groupings defined in 2.1)
Methods	12(a)	Describe all statistical methods, including those used to control for	p. 8 (Methods 2.3: Python 3.10; Spearman correlations; z-standardised

		confounding.	simple linear regression models)
Methods	12(b)	Describe any methods used to examine subgroups and interactions.	p. 8 (Methods 2.3: Analyses conducted separately for children, adolescents, adults)
Methods	12(c)	Explain how missing data were addressed.	p. 8 (Methods 2.3: Manual check; exclusion of incomplete key variables; no imputation)
Methods	12(d)	If applicable, describe analytical methods taking account of sampling strategy.	N/A (Convenience sampling used, no weighting required)
Methods	12(e)	Describe any sensitivity analyses.	p. 8 (Methods 2.3: Inspection for inconsistent response patterns and influential outliers)
Results	13(a)*	Report numbers of individuals at each stage of study.	p. 9 (Results 3.1: Total n=832; 382 children, 136 adolescents, 288 adults)
Results	13(b)	Give reasons for non-participation at each stage.	p. 9 (Results 3.1: Non-participation minimal; incomplete questionnaires or withdrawal of consent)
Results	13(c)	Consider use of a flow diagram.	N/A
Results	14(a)*	Give characteristics of study participants (e.g., demographic, clinical, social).	p. 9 (Results 3.1 and Table 1)
Results	14(b)	Indicate number of participants with missing data for each variable of interest.	p. 9 (Results: "missing data below 5% across all variables")
Results	15*	Report numbers of outcome events or summary measures.	pp. 9–10 (Results 3.2–3.4: MD adherence measures)
Results	16(a)	Give unadjusted estimates and, if applicable, confounder-adjusted estimates and their precision.	pp. 9–10 (Results: Standardised coefficients (Beta) and p-values presented)
Results	16(b)	Report category boundaries when continuous variables were categorized.	N/A
Results	16(c)	If relevant, consider translating estimates of relative risk into absolute risk for a meaningful time period.	N/A
Results	17	Report other analyses done—e.g., analyses of subgroups and interactions, and sensitivity analyses.	p. 10 (Results reported by age subgroups)
Discussion	18	Summarise key results with reference to study objectives.	p. 11 (Discussion: first paragraph)

Discussion	19	Discuss limitations of the study, taking into account sources of potential bias or imprecision.	p. 12 (Discussion: "Several limitations must be acknowledged...")
Discussion	20	Give a cautious overall interpretation of results considering objectives, limitations, etc.	pp. 11–13 (Discussion and Conclusions)
Discussion	21	Discuss the generalisability (external validity) of the study results.	p. 12 (Discussion: "...sample covered a wide range...")
Other info	22	Give the source of funding and the role of the funders for the present study.	p. 15 (Funding: PRIMA Programme, MICIU/AEI, EU)

*Give information separately for exposed and unexposed groups.

Note: An Explanation and Elaboration article discusses each checklist item and gives methodological background and published examples of transparent reporting. The STROBE checklist is best used in conjunction with this article (freely available on the Web sites of PLoS Medicine at http://www.plosmedicine.org/, Annals of Internal Medicine at http://www.annals.org/, and Epidemiology at http://www.epidem.com/). Information on the STROBE Initiative is available at www.strobe-statement.org.
